# Treating Alveolar Abscess

**Published:** 1899-10

**Authors:** 


Treating Alveolar Abscess.—When treating a case of blind abscess just commencing to swell, take a large raisin and divide it in two halves. Remove the seed, and warm it over a spirit lamp. Apply to gum, as a plaster, over affected root. Give patient saline laxative, and the following:

Iodide of potassium....................dr. ij.
Syr. sarsaparilla.......................oz. iij.

M. Sig. Teaspoonful three times a day before meals.

Of course, the canals of the roots should receive antiseptic treatment.

